# Cell culture-based production of defective interfering particles for influenza antiviral therapy

**DOI:** 10.1007/s00253-017-8660-3

**Published:** 2017-12-05

**Authors:** Milena A. Wasik, Luca Eichwald, Yvonne Genzel, Udo Reichl

**Affiliations:** 1 Bioprocess Engineering, Max Planck Institute for Dynamics of Complex Technical Systems, Sandtorstrasse 1, 39106 Magdeburg, Germany; 2 Bioprocess Engineering, Otto von Guericke University Magdeburg, Universitaetsplatz 2, 39106 Magdeburg, Germany

**Keywords:** Defective interfering particles, DI244 antiviral, Influenza A virus, Cell culture, Suspension cell line

## Abstract

Defective interfering particles (DIPs) lack an essential portion of the virus genome, but retain signals for replication and packaging, and therefore, interfere with standard virus (STV) replication. Due to this property, DIPs can be potential antivirals. The influenza A virus DIP DI244, generated during propagation in chicken eggs, has been previously described as a potential candidate for influenza antiviral therapy. As a cell culture-based manufacturing process would be more suitable to fulfill large-scale production needs of an antiviral and enables full process control in closed systems, we investigated options to produce DI244 in the avian cell line AGE1.CR.pIX in chemically defined suspension culture. With a DI244 fraction of 55.8% compared to STV, the highest DI244 yield obtained from 50 million cells was 4.6 × 10^9^ vRNA copies/mL at 12 h post infection. However, other defective genomes were also detected. Since these additionally produced defective particles are non-infectious, they might be still useful in antiviral therapies. In case they would interfere with quality of the final product, we examined the impact of virus seeds and selected process parameters on DI244 yield and contamination level with other defective particles. With a DI244 fraction of 5.5%, the yield obtained was 1.7 × 10^8^ vRNA copies/mL but now without additional defective genomes. Although the DI244 yield might be decreased in this case, such controlled manufacturing conditions are not available in chicken eggs. Overall, the application of these findings can support design and optimization of a cell culture-based production process for DIPs to be used as antivirals.

## Introduction

Influenza viruses are enveloped viruses of the *Orthomyxoviridae* family with a segmented genome that consists of eight negative-stranded RNA segments. Influenza viruses can cause respiratory illness known as flu. Annual flu epidemics worldwide are estimated by the WHO to result in about 3 to 5 million cases of severe illness, and about 250,000 to 500,000 deaths. The most effective way to prevent flu is vaccination, but influenza vaccines do not always match the strains that are currently circulating. Two classes of antiviral drugs against influenza are currently available, to which however, virus resistance has become more and more frequent (Mc Mahon and Martin Loeches 2017, WHO 2017]. This raises the need for new antiviral drugs.

The use of defective interfering particles (DIPs), which reduce infectious load and stimulate the adaptive and innate immunity (Scott et al. 2011a), is one such new approach for an influenza antiviral therapy. One candidate is DI244, described by Dimmock et al. (Dimmock et al. 2008), which differs from infectious influenza A virus only by a single internal deletion in the largest genomic segment 1 that codes for the polymerase basic protein 2 (PB2). The DI244 RNA comprises 395 nt instead of 2341 nt, but contains the termini of the RNA that carry the replication and packing signals essential for the DIPs to be propagated and packaged. Due to the defective virus genome, DI244 and other DIPs are not able to replicate on their own, but require the presence of a completely functional standard virus (STV), which provides the missing protein(s). DIPs interfere with the replication of STV in co-infected cells, and thus, can efficiently reduce the infectious particle production of some influenza strains (Frensing et al. 2014). In particular, it was shown that DI244 protects mice and ferrets from lethal infection caused by a number of different influenza A viruses, an influenza B virus strain, and a murine pneumovirus strain, suggesting further development as a broad-acting antiviral (Dimmock et al. 2012, Scott et al. 2011b, Easton et al. 2011).

Until now, DI244 has been grown in embryonated chicken eggs (Dimmock et al. 2008). While egg-based influenza vaccine production is well understood and produces consistent results, it has several disadvantages, i.e., it is poorly scalable, involves the possibility of bacterial contamination that necessitates the addition of antibiotics, and some people suffer from allergic reactions to egg components that may be present in the product (Minor et al. 2009, Perdue et al. 2011, Chung 2013). Influenza virus propagation in cell culture overcomes these problems (WHO 1995). Moreover, cell culture-based production can be initiated without long lead times and takes place in a completely closed and aseptic environment with full control of cultivation conditions, substrates, and quality. Furthermore, it was shown for one influenza vaccine that it contained substantial amounts of defective RNA that arose during production in chicken eggs (Gould et al. 2017). This can be true for other influenza vaccines as well. Whether vaccine production without accumulation of defective RNAs is possible in cell culture has not been reported yet.

In this study, we investigated the replication of DI244 in a designer cell line established to replace primary chicken cells in vaccine production (Jordan et al. 2009). The cell line AGE1.CR.pIX originates from the Muscovy duck, is well characterized, and has been reported to support fast propagation of influenza viruses (Lohr et al. 2009). Suspension growth of this cell line in a chemically defined medium allows the development of easily scalable processes, fulfilling all requirements in state-of-the-art vaccine manufacturing. Usually, the formation of DIPs is unwanted in influenza vaccine manufacturing, since DIPs can lower the virus yields (Frensing et al. 2014). Therefore, low multiplicity of infection (MOI) conditions are typically chosen for virus propagation. Little is known however, which conditions are required for cell culture-derived production of high-yield DIP preparations for animal and clinical studies, and for establishment of commercial manufacturing processes.

In this study, we characterized specific properties of a virus seed used for DI244 production in AGE1.CR.pIX cells and the impact of cultivation conditions on DI244 yield and contamination level with additional defective genomes, i.e., the impact of MOI and trypsin activity. In addition, we have established a RT-qPCR assay which enables the quantification of DI244 and STV copy numbers based on segment 1, which cannot be monitored by other titration methods, i.e., TCID_50_ and PFU assay (infectious virus particles) or HA assay (total number of virus particles). Based on small-scale experiments, we propose a cell culture-based process for DIPs generation, specifically DI244, for manufacturing of this new class of antivirals.

## Materials and methods

### Cells

The cell line AGE1.CR.pIX was provided by ProBioGen AG and cultivated in 125 mL vented and baffled shaking flasks (wv, working volume 50 mL) at 185 rpm, 37 °C, and 5% CO_2_. The chemically defined CD-U3 medium (Biochrom/Merck) was supplemented with 2 mM glutamine (Sigma), 2 mM alanine (Sigma), and 10 ng/mL recombinant insulin-like growth factor (LONG^3^ IGF, Sigma). Cells were passaged twice a week by dilution to the starting cell concentration of 8 × 10^5^ cells/mL using fresh medium.

Adherent MDCK cells (ECACC, #84121903), used for the innocuity test (see Results, UV irradiation for inactivation of standard virus) and TCID_50_ assay (see Measurement of virus titers), were maintained in T75 flasks containing 50 mL GMEM (GIBCO) supplemented with 10% (*v*/*v*) fetal calf serum (PAN Biotech) and 1% (*v*/*v*) peptone (Lab M) at 37 °C in a 5% CO_2_ atmosphere.

### Viruses

The egg-derived DI244/PR8 virus, previously described (Dimmock et al. 2008) and here referred to as DI244/STV, was provided by Nigel J. Dimmock from the University of Warwick (UK). For adaptation to AGE1.CR.pIX cells and to generate virus seed for subsequent experiments, DI244/STV was serially passaged using a trypsin activity of 3 × 10^−6^ U/cell. The first passage was conducted by directly infecting cells with 50 μL of DI244/STV. The resulting virus showed a TCID_50_ titer of 7.2 × 10^4^ infectious virions/mL and a HA titer of 1.37 log HAU/100 μL at 12 hpi. For the second passage, MOI 10^−3^ (based on TCID_50_ assay) was used and the virus was harvested at 24 hpi. The third passage was performed at MOI 10^−6^ and resulted in the DI244 working virus seed, which showed a TCID_50_ titer of 5.5 × 10^5^ infectious virions/mL and a HA titer of 1.45 log HAU/100 μL at 24 hpi. The fourth passage was conducted at MOI 10^−8^ and resulted in the DIP-free STV working virus seed, which showed a TCID_50_ titer of 2.1 × 10^6^ infectious virions/mL, a HA titer of 1.82 log HAU/100 μL, and 6.8 × 10^9^ STV vRNA copies/mL by RT-qPCR at 48 hpi. No defective genomes including DI244 were detected by RT-PCR. In addition, a STV-free DI244 seed was produced by UV irradiation (see below) of the DI244 working virus seed.

### UV irradiation

UV irradiation to destroy the full-length segments of STV (Dimmock et al. 2008) for co-infection experiments (see Results, UV irradiation for inactivation of standard virus) was performed in 12-well plates with a surface area of 4 cm^2^. A volume of 350 μL per well (liquid layer of approx. 1 mm) of the DI244 working virus seed was exposed for 8.5 min to UV light (254 nm) in the PCR Workstation Pro (VWR Peqlab) with a distance of about 65 cm to the two UV tubes (each 25 W).

### Infections

After inoculating 8 × 10^5^ AGE1.CR.pIX cells/mL, the cells were grown 3–4 days in shaker flasks. Cells were diluted without any washing steps to a cell concentration of 1 × 10^6^ cells/mL in a volume of 50 mL using fresh medium. Calculated volumes of virus seed for the desired MOIs between 10^−1^ and 10^−8^ (as indicated for each experiment), based on the TCID_50_ assay (see Measurement of virus titers), and trypsin stock solution (500 units/mL, GIBCO) for the desired trypsin activity (as indicated for each experiment) were added directly to the cell suspension. Virus samples were collected at 36 h post infection (hpi) if not indicated otherwise. Samples were centrifuged at 300g for 5 min to remove cells and supernatant was stored at − 80 °C until RNA isolation.

For the innocuity test, confluent cultures of adherent MDCK cells were washed twice with PBS. 1 mL of UV-irradiated DI244 working virus seed was added to the cells in 50 mL serum-free GMEM (GIBCO) containing 1% (*v*/*v*) peptone (Lab M) and 5 U/mL trypsin (GIBCO, #27250-018). Virus samples were collected at 72 hpi. Samples were centrifuged at 300g for 5 min and the supernatant was stored at − 80 °C until HA assay.

### Measurement of virus titers

Infectious virus particle concentrations were measured using a tissue culture infectious dose 50 (TCID_50_) assay by titration of supernatant samples on MDCK monolayers as described previously (Genzel and Reichl 2007). Titers were expressed as infectious virions/mL.

Total virus particle concentrations were determined using a hemagglutination assay as described previously (Kalbfuss et al. 2008). Titers are expressed as log_10_ HA units per test volume (log HAU/100 μL).

### Segment-specific RT-PCR for detection of defective genomes

RNA was isolated from 150 μL supernatant using the NucleoSpin RNA Virus Kit (Macherey-Nagel) according to the manufacturer’s instructions.

The reverse transcription (RT) of isolated RNA to cDNA as well as the amplification of all eight influenza virus genome segments with segment-specific primers for detection of defective genomes was performed as described previously (Frensing et al. 2014). The RT-PCR products were directly analyzed on a 1% agarose gel using electrophoresis.

### Segment-specific RT-qPCR for DI244 and STV quantification

To determine viral RNA (vRNA) copy numbers, a reverse transcription quantitative PCR (RT-qPCR) was performed using RNA reference standards for the targeted RNA as described previously (Frensing et al. 2014). Briefly, pUC19 plasmids with inserted full-length segment 1 (FL1) or DI244 cDNA were cloned, respectively. Plasmids were subjected to PCR with the primer pair Seg-1-Uni-for (AGCGAAAGCAGGTCAATTAT) and Seg-1-Uni-T7-rev (TAATACGACTCACTATAGGGAGTAGAAACAAGGTCGTTTTTAAAC) to introduce a T7 promoter sequence in the desired orientation into the PCR product. PCR products were in vitro transcribed to vRNA. Absolute vRNA copy numbers were determined as described previously (Frensing et al. 2014). In brief, a 10-fold dilution series of the corresponding vRNA reference standards and the undiluted isolated RNA were reverse transcribed with the polarity-specific and tagged primer Seg-1-tagRT-for (ATTTAGGTGACACTATAGAAGCGAGCGAAAGCAGGTCAATTATATTC). To distinguish DI244 from FL1, primer sets were used in qPCR, with one primer binding to the junction region of the defective segment, DI244-realtime-rev (GGAATCCCCTCAGTCTTC), and to the region of FL1, which is deleted from DI244, respectively, FL1-realtime-rev (CATTTCATCCTAAGTGCTGG), and one primer binding to the tag, which was prior to that introduced in reverse transcription, vRNA-tagRealtime-for (ATTTAGGTGACACTATAGAAGCG). The Rotor-Gene SYBR Green PCR Kit (Qiagen) and the Rotor-Gene Q real-time PCR cycler (Qiagen) were used for quantification following the manufacturer’s recommendations. Viral RNA copy numbers in the samples (isolated RNA; see above) were calculated based on the vRNA reference standards with linear regression. Since the qPCR primers bind to extracellular vRNA and theoretically only one copy of vRNA is packed per virion, the qPCR data is equivalent to the total number of virus particles. Standard curves showed strong linear correlations (> 0.99) over eight orders of magnitude for DI244 and seven orders of magnitude for FL1, respectively (data not shown). Amplification efficiency was between 98 and 108%. The lowest concentration at which linearity was retained in the standard curve was 2.2 × 10^3^ DI244 copies/μL and 3.8 × 10^3^ FL1 copies/μL, respectively. The inter-assay coefficient of variation for the copy numbers was 6.4% for DI244 vRNA and 5.1% for FL1 vRNA, respectively.

## Results

### Generation of a defined DI244 working virus seed for cell culture experiments

**Fig. 1:**
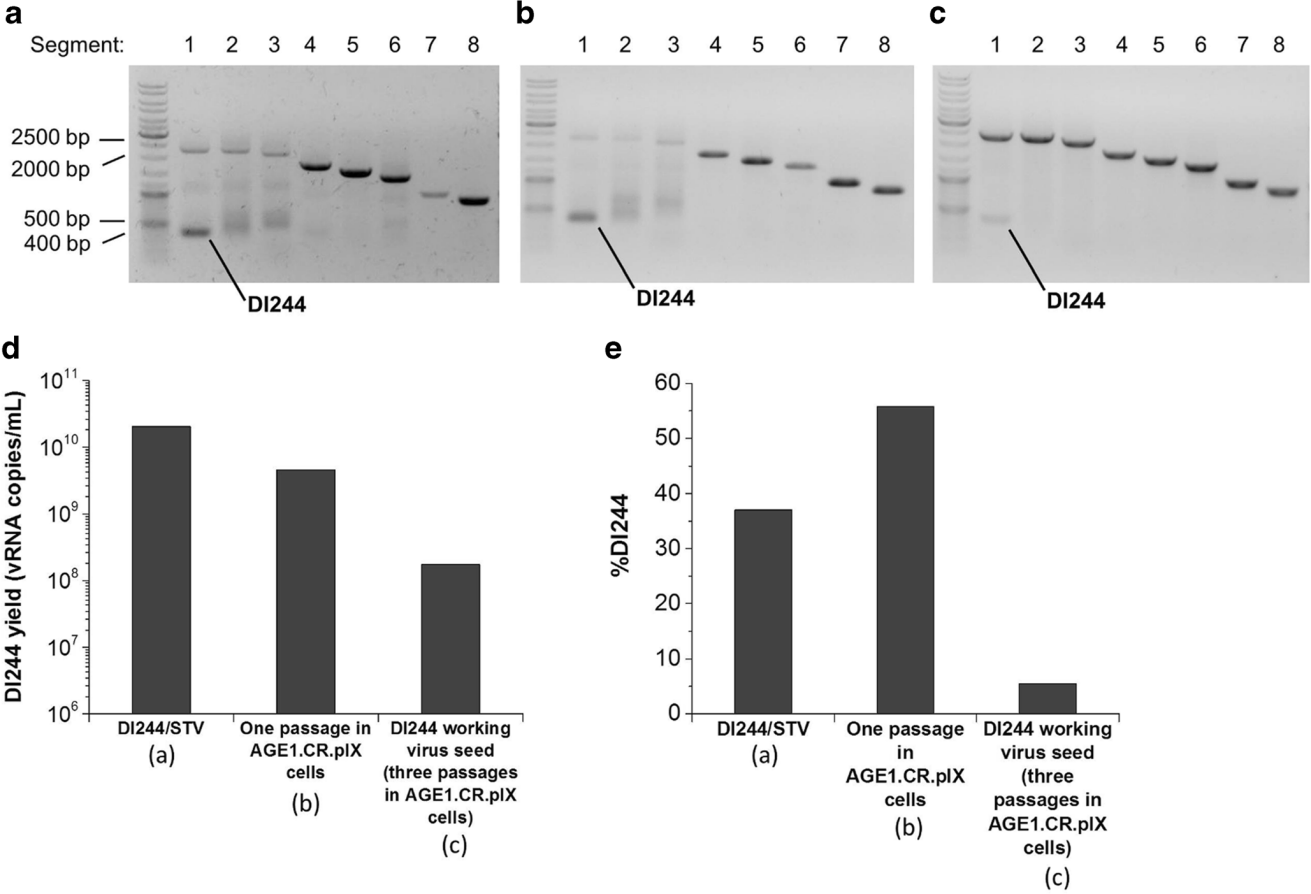
Gel electrophoretic analysis of segment-specific RT-PCR to detect defective influenza virus genomic RNAs (all eight influenza genome segments were amplified) and quantification of DI244 by RT-qPCR. **a** RT-PCR of DI244/STV. **b** RT-PCR of DI244/STV after one passage in AGE1.CR.pIX cells; AGE1.CR.pIX cells were infected with 50 μL DI244/STV and supernatant was harvested at 12 hpi. **c** RT-PCR of DI244 working virus seed after three passages in AGE1.CR.pIX cells. Supernatant was harvested at 24 hpi. **d** Copy numbers of DI244 vRNA in virus material obtained from one egg (10 mL assumed for estimation of yield) or 50 × 10^6^ AGE1.CR.pIX cells in 50 mL working volume (corresponding to samples from Fig. 1 a, b, and c). Measurements were performed in triplicates (error too small to be shown). **e** Percentage of DI244 in virus material shown in Fig. 1 d, measured by RT-qPCR (error too small to be shown)

Influenza virus propagation in embryonated chicken eggs and animal cells can favor the generation of various defective genomes. Indeed, besides DI244, we detected multiple uncharacterized defective genomes in segments 1, 2, 3, 4, and 6 in the DI244/STV material provided (Fig. 1a). To generate a better defined working virus seed for cell culture experiments, we investigated if these additional defective genomes in DI244/STV can be depleted after several replication rounds in cell culture. Initially, we chose the Madin-Darby Canine Kidney (MDCK) cell line MDCK.SUS2, adapted to serum-free suspension growth, as a substrate, since MDCK cells support the replication of influenza virus to high titers and are already licensed as host cells for influenza vaccine manufacturing (Peschel et al. 2013, Hegde 2015). However, even after several passages of DI244/STV in MDCK.SUS2 cells, additional defective genomes of segments 1, 2, 3, 4, and 6 were still detected by RT-PCR (data not shown). Since the generation of defective particles is cell dependent (De and Nayak 1980, Crumpton et al. 1981), we tested different cell lines, e.g., the cell line AGE1.CR.pIX, which was specifically designed for vaccine manufacturing in chemically defined media (Jordan et al. 2009). Moreover, the AGE1.CR.pIX suspension cell line was shown to propagate influenza virus efficiently (Lohr et al. 2012). We infected AGE1.CR.pIX cells with 10-fold dilutions of DI244/STV. In the virus material harvested after one passage in AGE1.CR.pIX cells, no defective genomes of segments 4 and 6 were detected by RT-PCR (Fig. 1b). By subjecting this material to two more passages in AGE1.CR.pIX cells, we obtained a DI244 working virus seed without noticeable defective genomes besides DI244 (Fig. 1c). Since this RT-PCR can only serve as a visual quality control, the newly established RT-qPCR was used to determine DI244 vRNA copy numbers in the samples.

Assuming an egg volume of 10 mL (allantoic fluid), the egg-derived virus material showed the highest DI244 vRNA concentration with 20.5 × 10^9^ copies/mL (Fig. 1d). The virus harvested after one passage from 50 × 10^6^ AGE1.CR.pIX cells showed a DI244 yield of 4.6 × 10^9^ copies/mL, which decreased by about one order of magnitude after three passages to 1.7 × 10^8^ copies/mL for the resulting DI244 working virus seed (Fig. 1d). The percentage of DI244 differed significantly for all DI244 containing virus materials. It started with 37.0% in the egg-derived material, increased to 55.8% after one passage in cell culture, but decreased to 5.5% for the working virus seed free of additional defective genomes (Fig. 1e).

### Screening for optimal cultivation conditions for DI244

For a systematic characterization of the impact of selected process conditions on DI244 yield and on the generation of additional defective genomes, we varied both MOI and trypsin activity, which are critical parameters in influenza vaccine production (Genzel and Reichl 2009). For all experiments, the DI244 working virus seed (Fig. 1c) was used. Since defective genomes are more likely to occur at high MOIs (Heldt et al. 2013), we started with a low MOI (10^−5^) to reduce the risk of *de novo* generation of additional defective genomes and increased MOI stepwise. The addition of trypsin at time of infection is required for hemagglutinin cleavage to facilitate virus infection. A trypsin activity of 10^−6^ U/cell was previously shown to be the most effective in influenza virus production in AGE1.CR.pIX cells (Lohr et al. 2012). Therefore, in addition to MOI, trypsin activities in the range 10^−5^ to 10^−7^ U/cell were also tested. Furthermore, supernatants from four different harvesting time points were characterized by RT-PCR. The harvests were analyzed for DI244 and possible contamination with other defective genomes in segments 1, 2, and 3, since defective RNAs are more likely to be generated from polymerase coding segments (Nayak et al. 1985).

**Fig. 2:**
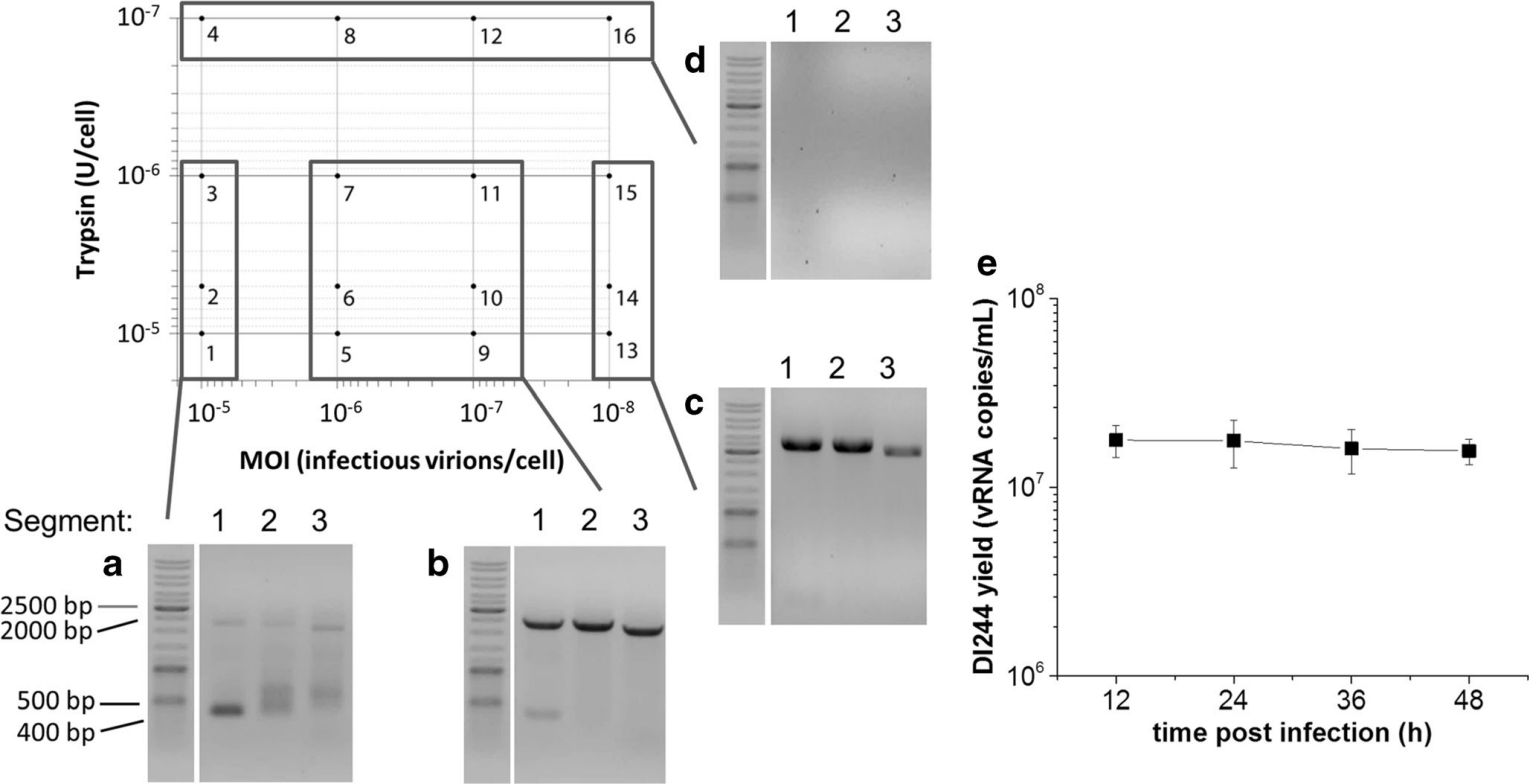
Trends observed during infection of AGE1.CR.pIX cells with the DI244 working virus seed for different MOIs and trypsin activities. **a** (#3) Infections at MOI 10^−5^ and trypsin activities in the range 10^−5^ and 10^−6^ U/cell showed a strong band for DI244 as well as de novo generation of defective genomes in segments 2 and 3. **b** (#7) Infections at the MOIs 10^−6^ and 10^−7^ and trypsin activities in the range 10^−5^ and 10^−6^ U/cell did not favor the generation of additional defective genomes. **c** (#15) Infections at MOI 10^−8^ and trypsin activities in the range 10^−5^ and 10^−6^ U/cell resulted in the loss of DI244. **d** (#8) Infections with a trypsin activity of 10^−7^ U/cell showed no viral RNA bands by RT-PCR for all MOIs. Samples, collected at 36 hpi, are shown. Numbers above the gel pictures represent the segment numbers; numbers associated with each gel represent the specific experimental condition. **e** RT-qPCR analysis of vRNA of DI244 () over time at an MOI of 10^−5^ and a trypsin activity of 10^−6^. Error bars indicate standard deviation of three independent biological experiments

We were able to observe three trends after infection of AGE1.CR.pIX cells with the DI244 working virus seed at trypsin activities in the range 10^−5^ to 10^−6^ U/cell: A variety of additional defective genomes in segments 2 and 3 were generated at MOI 10^−5^ (Fig. 2a). No additional defective genomes were generated at MOI 10^−6^ and 10^−7^ (Fig. 2b). Infections at MOI 10^−8^ resulted in the loss of DI244 (Fig. 2c). No virus replication was observed for all MOIs at a trypsin activity of 10^−7^ U/cell (Fig. 2d). Obviously, this trypsin activity was too low for successful virus uptake and initiation of virus replication. We screened further under conditions shown in Fig. 2b and measured the DI244 copy number by RT-qPCR. Interestingly, the DI244 copy numbers were stable, corresponding to the initial DI244 copy number of the DI244 working virus seed (1.7 × 10^8^ copies/mL). The highest copy number was achieved 12 hpi with only a slight decrease over the next 36 h (Fig. 2e). Overall, this data suggests that the MOIs and trypsin activities should be adjusted to the range 10^−6^ to 10^−7^ and 10^−5^ to 10^−6^, respectively, for cell culture-derived DI244 production.

### UV irradiation for inactivation of standard virus

**Fig. 3:**
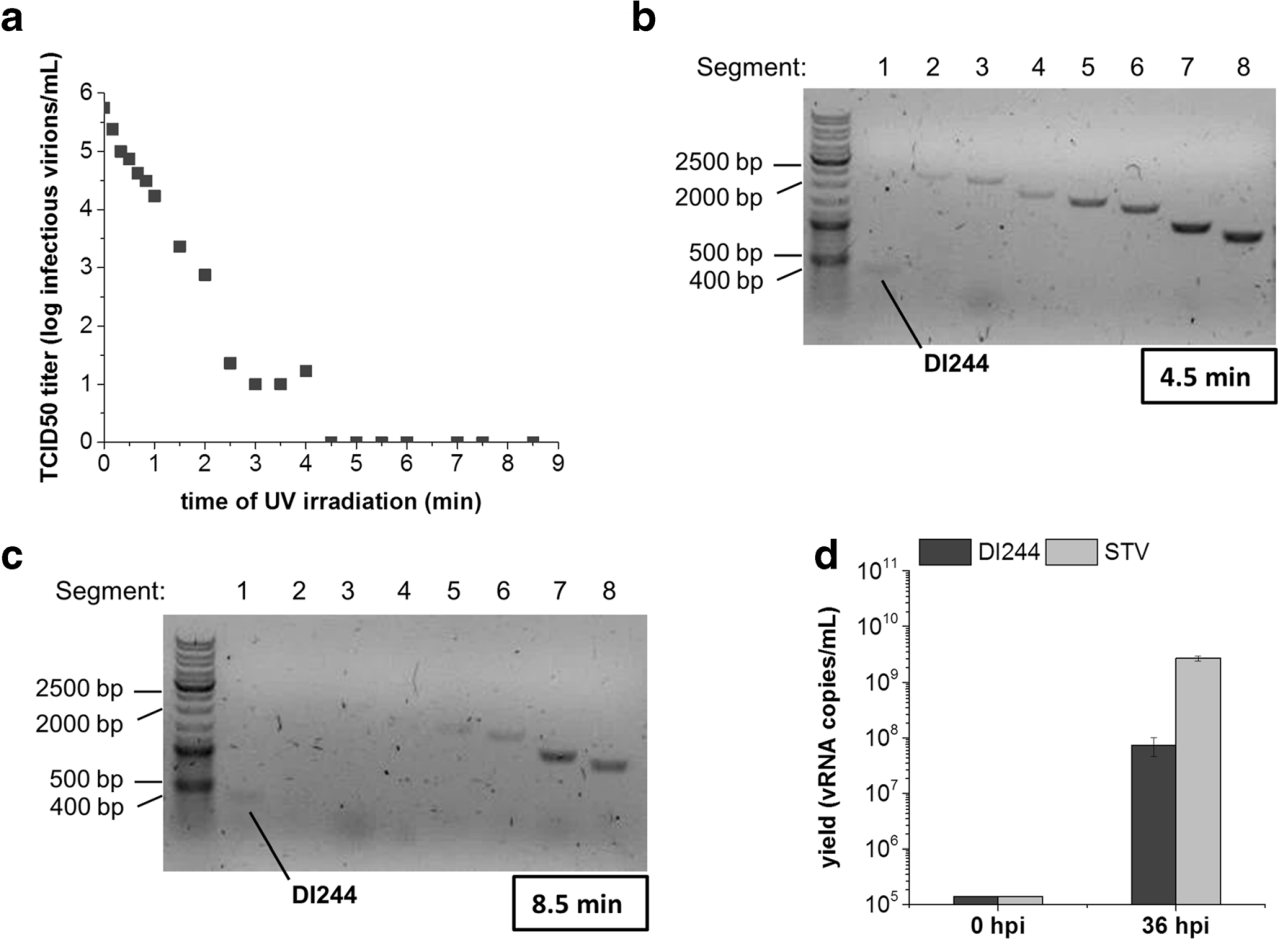
Inactivation of DI244 working virus seed by UV irradiation. **a** Decrease in TCID_50_ with UV irradiation time. Samples, UV irradiated for 4.5 min or 8.5 min, respectively, were measured in independent triplicates (error too small to be shown). **b** Gel electrophoretic analysis of segment-specific RT-PCR of all eight influenza genomic RNAs (one to eight) and DI244 RNA in the DI244 working virus seed after UV irradiation for 4.5 min. **c** Gel electrophoretic analysis of segment-specific RT-PCR of all eight influenza genomic RNAs (one to eight) and DI244 RNA in the DI244 working virus seed after UV irradiation for 8.5 min. **d** DI244 activity in AGE1.CR.pIX cells after UV irradiation for 8.5 min and subsequent infection with STV (MOI 10^−4^, ration 1:1). At 36 hpi, an increase in STV (light gray) as well as DI244 (dark gray) vRNA copy numbers was observed by RT-qPCR. Error bars indicate standard deviation of three independent biological experiments

After UV irradiation, infectivity of STV was measured by TCID_50_ assay for a range of UV exposure times (10 s to 8.5 min). The specific inactivation rate of this first order reaction was 3.1 min^−1^ (linear range 0.33 to 2.0 min). Complete loss of infectivity (TCID_50_ titer below the detection limit) was observed for UV exposure times of 4.5 min and above (Fig. 3a). Further RT-PCR analysis showed that FL1 was not amplified after 4.5 min of UV irradiation (Fig. 3b), indicating degradation of FL1. To ensure that no replication competent virus was present after UV irradiation, the innocuity of STV was tested for two serial passages in MDCK cells by monitoring HA titers. The necessary UV exposure time to ensure complete inactivation of STV was identified as 8.5 min (data not shown). For this time point, RT-PCR showed that all larger segments (2341 to 1778 nt) FL1, 2, 3, and 4 were destroyed (Fig. 3c), confirming again complete inactivation of STV. In addition, it was tested, whether DI244 replication was affected by UV irradiation for 8.5 min. Therefore, AGE1.CR.pIX cells were co-infected with DIP-free STV working virus seed (MOI 10^−4^, i.e., 1.4 × 10^5^ STV vRNA copies/mL) and the same concentration of UV irradiated (8.5 min) DI244. At 36 hpi, an increase in vRNA copy numbers was observed for STV and DI244 by RT-qPCR (Fig. 3d). Hence, DI244 RNA was not significantly affected by UV irradiation due to its smaller genome size compared to the full-length infectious viral genome. This finding corresponds to the RT-PCR gel that shows a band for DI244 even after 8.5 min of UV irradiation (Fig. 3c).

### Impact of initial DI244/STV ratio on DI244 yield

**Fig. 4:**
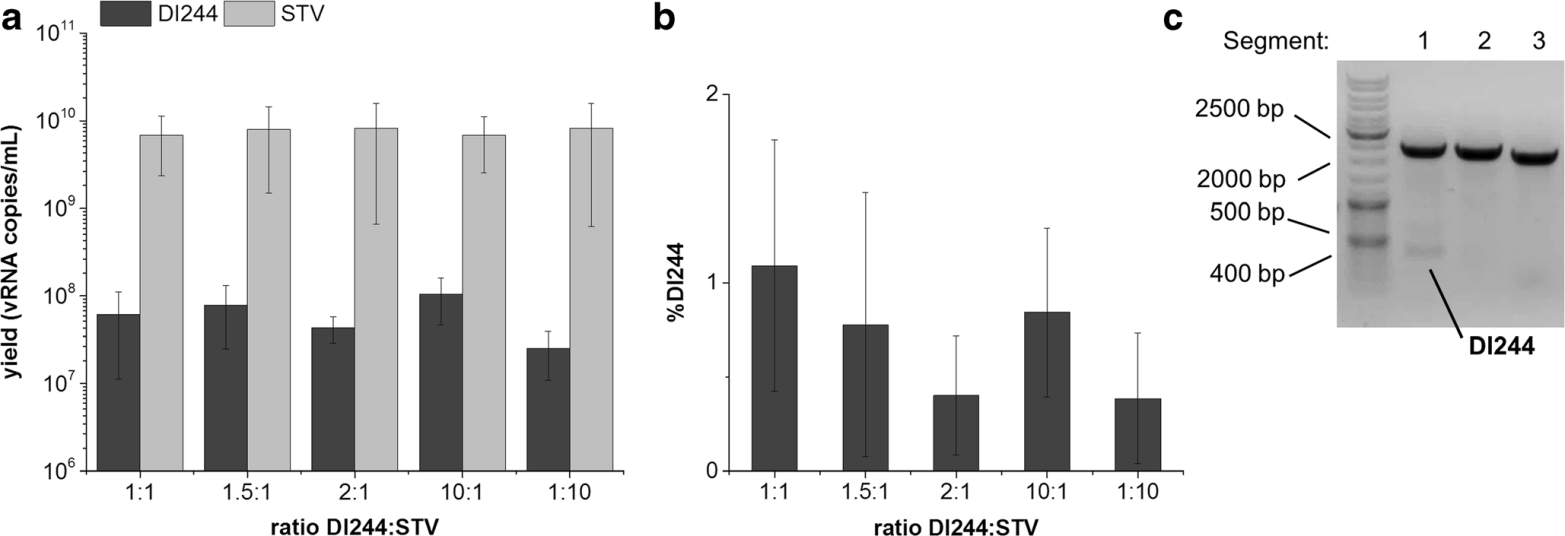
**a** DI244 (dark gray) and STV (light gray) content and **b** percentage of DI244 after infection of AGE1.CR.pIX cells with the ratios 1:1, 1.5:1, 2:1, 10:1, 1:10 DI244/STV at MOI 10^−6^ using a trypsin activity of 10^−6^ U/cell. Samples were collected at 36 hpi. **c** Gel picture of RT-PCR analysis representative for all tested ratios. Error bars indicate standard deviation of three independent biological experiments

By analyzing the effect of MOI on DI244 propagation, we found that MOIs in the range 10^−6^ to 10^−7^ showed the most promising results as the generation of additional defective genomes could be prevented (Fig. 2b). However, the fraction of DI244 was rather low with < 2%. It has to be taken into account, however, that the infection was performed with the DI244 working virus seed which had already a low DI244 fraction of 5.5% (Fig. 1e). Most likely, this resulted in poor replication conditions of DI244 compared to STV. To determine the impact of the DI244/STV ratio of working seeds on DI244 yield in virus harvests, we infected cells with the same amount or an excess of DI244 over STV. For this experiment, we produced a DIP-free STV working virus seed using a MOI of 10^−8^ and a trypsin activity of 3 × 10^−6^ U/cell, respectively, and a STV-free DI244 stock by UV irradiation. Then, we infected AGE1.CR.pIX cells at a MOI of 10^−6^ with STV and added DI244 in the vRNA copy number ratios 1:1, 1.5:1, 2:1, 10:1, and 1:10, determined by RT-qPCR. Higher DI244/STV ratios were not tested, since this could lead to interference of DI244 with STV, and thus, inhibition of DIP replication, resulting in reduced yields (Scott et al. 2011a, Akkina et al. 1984, Stauffer Thompson et al. 2009). For harvests at 36 hpi and all ratios tested, DI244 yields were in the same order of magnitude at about 2.5 to 10.4 × 10^7^ vRNA copies/mL (Fig. 4a) with a DI244 fraction not exceeding 1.1% (Fig. 4b). No additional defective genomes were detected by RT-PCR (Fig. 4c). This is according to the results shown in Fig. 2b, where we showed that a low MOI is preventing the generation of additional defective genomes, but does not benefit DI244 production.

### Impact of MOI for infections with same DI244/STV ratio

**Fig. 5:**
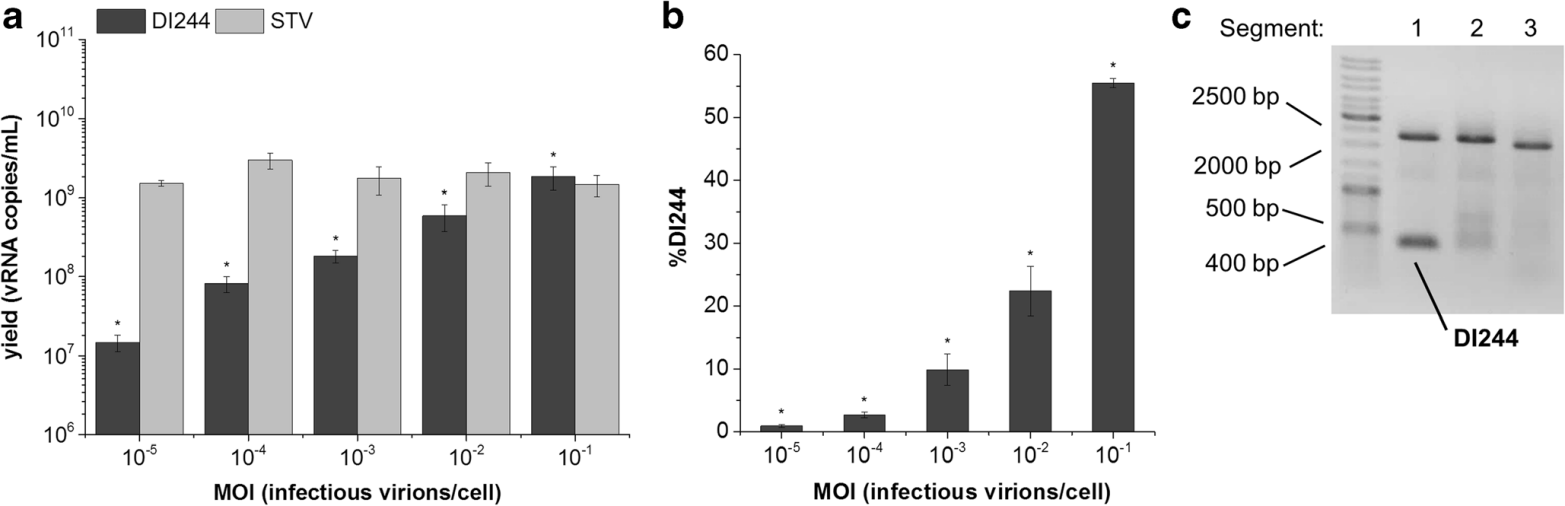
**a** Yield and **b** percentage of DI244 (dark gray) and STV (light gray) after co-infection of AGE1.CR.pIX cells with ratio of 1:1 and different MOI using a trypsin activity of 10^−6^ U/cell. Samples were collected at 36 hpi. **c** Gel picture of RT-PCR analysis representative for all tested MOI. Error bars indicate standard deviation of three independent biological experiments (*p < 0.05 for DI244 at all tested MOI by Kruskal-Wallis ANOVA)

To better characterize DI244 replication in cell culture and evaluate options to increase the DI244 content, we quantitatively analyzed the MOI dependency on DI244 yield. Using the virus seeds generated for the experiments above, we infected AGE1.CR.pIX cells with a constant DI244/STV ratio of 1:1, increased the MOI stepwise, and measured the DI244 vRNA concentration by RT-qPCR. A constant DI244/STV ratio of 1:1 was chosen to give both DI244 and STV the same chance of replication. For 36 hpi, we observed that the DI244 yield as well as the DI244 fraction increased proportionally to MOI (Fig. 5a, b). However, at MOIs 10^−5^ to 10^−2^, the STV yield was higher. A maximum of 1.8 × 10^9^ DI244 vRNA copies/mL was obtained for MOI 10^−1^, where the DI244 showed a slight replication advantage over STV (Fig. 5a). Overall, we were able to increase the DI244 yield by two orders of magnitude and the DI244 fraction to 54.5%, respectively. However, at all tested MOIs, defective genomes were generated in segments 1 (at about 1500 bp), 2, and 3 in addition to DI244 (Fig. 5c).

## Discussion

### Cell culture-based DI244 production as an alternative to eggs

Antiviral drug resistance of influenza viruses is an increasing problem and the demand for new options for antiviral therapy rises. DI244 was previously described to be a good candidate for blocking influenza virus infections (Dimmock et al. 2008). It was generated by a reverse genetic approach and passaged in embryonated chicken eggs to increase DI244 yields, which resulted in generation of numerous additional defective genomes. As an alternative, cell culture-based processes, which have been established over the last years, could be used for large-scale production in a completely contained and fully controlled environment. We therefore investigated options to generate suitable DI244 working virus seeds and established small-scale DI244 production in AGE1.CR.pIX suspension cells, which were specifically designed for vaccine manufacturing in chemically defined media.

### Comparison of DI244 yields in eggs and cell culture

By replicating the egg-derived DI244/STV in AGE1.CR.pIX cells, we were able to eliminate defective genomes of segments 4 and 6. Also, the fraction of DI244 increased from 37.0% for the egg-derived to 55.8% for the cell culture-derived harvests. Assuming an egg volume of 10 mL (allantoic fluid), the egg-derived virus material showed with 20.5 × 10^9^ copies/mL a higher DI244 vRNA concentration then the virus harvested after one passage in AGE1.CR.pIX cells with 4.6 × 10^9^ copies/mL. However, the cultivation was performed in small-scale shaking flasks with the relatively low concentration of 1 × 10^6^ AGE1.CR.pIX cells/mL. This resulted in a cell-specific yield of 4.6 × 10^3^ DI244 vRNA copies/cell. In fully controlled bioreactors, however, AGE1.CR.pIX cells can be infected at concentrations up to 10 × 10^6^ cells/mL (Genzel et al. 2014). Thus, a 1 L bioreactor could yield 46 × 10^9^ DI244 vRNA copies/mL within 1 or 2 days post infection. One clinical dose for mice has been reported to contain about 3.4 × 10^9^ DI244 copies (Dimmock and Easton 2015). Thus, a 1 L bioreactor yield could be sufficient for providing 13.5 mouse doses per mL or a total of about 13,529 mouse doses. To gain the same yield, 244 fertilized eggs would be necessary, since an egg yields about six doses per mL. Therefore, cell culture-based production is a valid alternative to egg-based manufacturing processes and could be even superior to egg-based production. However, whether our findings are 1:1 scalable needs further investigation. In addition, to further improve DI244 yields, high-cell-density cultures could be implemented. For example, in alternating tangential flow perfusion systems, up to 50 × 10^6^ cells/mL have been described for growth of AGE1.CR.pIX cells and subsequent influenza A virus production (Genzel et al. 2014). Also, other production cell lines should be tested, since the rates of influenza A virus replication as well as DIP generation vary greatly among different cell lines (De and Nayak 1980, Crumpton et al. 1981).

### Quantification of DI244 and dose input by RT-qPCR

Although Dimmock and Easton specified DI244 copy numbers required for protection of mice, they used a specific DI244 virus protein mass for inoculation (Dimmock et al. 2008, Dimmock and Easton 2015). However, the estimation of DI244 input using protein concentration allows only a rough adjustment of the dose. Based on the established RT-qPCR assay, a more precise method for calculation of dose input for animal and human clinical trials is now available. Furthermore, this assay is also crucial for monitoring cell culture-based DI244 production and for establishment of mathematical models. Since virus titration assays are probably influenced by the presence of DIPs, and the TCID_50_ titers of DI244 preparations are relatively low, the calculated volume for inoculation of a bioreactor with a specific MOI could be overestimated. Adjusting the virus input by DI244 copy numbers would be a more reliable method for DI244 virus seed characterization and for monitoring of production.

### Impact of selected process parameters and virus seeds on DI244 yield

In a first step towards the design of DIP production processes, we examined the impact of the working virus seed as well as selected process parameters on DI244 yields. Among others, different ratios of DI244 to STV were tested with a constant MOI. To adjust these ratios, DI244 without other defective particles was used to reduce the risk of unspecific effects from additional defective genomes. Also, we chose a very low MOI of 10^−6^ to avoid the generation of defective particles other than DI244 (Fig. 2b). We observed that the initial ratio DI244/STV in the virus seed did not play a role in increasing the DI244 yield and fraction (Fig. 4a, b). However, at a MOI as low as 10^−6^, stochastic effects are likely, such as a low probability of co-infection of cells with DI244 and STV at first round(s) of replication. Furthermore, it was shown that up to 90% of influenza A virus-infected cells can fail to release infectious progeny at low MOI (Brooke et al. 2013). If such infections result mostly in non-productive progeny STV, DI244 cannot hijack (enough) FL1/PB2 for replication and preferential synthesis of DIPs as discussed previously (Stauffer Thompson et al. 2009, Dimmock and Easton 2015, Nayak et al. 1985). For this reason, we tested higher MOIs with a constant ratio of 1:1. We observed that the DI244 yield and fraction increased proportionally to MOI (Fig. 5a, b). The highest tested MOI of 10^−1^, where DI244 showed a slight replication advantage compared to STV, led to a sufficient DI244 yield comparable to the yield from one passage in AGE1.CR.pIX cells (Fig.1d, 5a). For large scale DI244 manufacturing, however, this would require the use of relatively large volumes of virus seeds and render production more complex or even impossible. Most likely, the use of MOIs in the range 10^−4^ to10^−2^ would be feasible and further studies should be performed with other DI244/STV ratios to optimize infection conditions. However, at process conditions relevant for the manufacturing of DI244 antivirals, we also detected additional defective genomes in the harvests. As relatively high MOIs were required for obtaining reasonable yields, de novo generation of defective particles could not be avoided. This seems also true for egg-based antiviral production systems. Therefore, a compromise between contamination with other defective particles, yield, and virus seed volume used for initiation of infection has to be found. Whether the accumulation of such additional defective genomes is a disadvantage or not for the treatment of animals or humans cannot be decided at the moment. However, no adverse effects have been reported after giving recipients an influenza vaccine which contained substantial amounts of defective RNAs (Gould et al. 2017). Though the study suggests that defective RNAs do not present a health problem, it would be of considerable public interest to investigate the effect of defective RNAs in influenza vaccines. In case there is no negative impact on antiviral safety and efficacy, both cell culture and egg-based production systems could be used for industrial large-scale manufacturing.

### UV irradiation for inactivating STV in DI244 manufacturing

For the use as an antiviral, infectious STV contaminating the produced DI244 harvests needs to be eliminated. Chemical inactivation is not possible, since it would also destroy DI244 RNA. Similar to egg-based DI244 production, UV irradiation of cell culture-derived DI244 could be an option to obtain a safe product, since it completely inactivates STV while leaving DI244 intact for intracellular replication. For the inactivation method used here, innocuity was demonstrated for STV containing samples after irradiation for 8.5 min. However, regarding the establishment of large-scale production processes, it has to be taken into account that there is only very limited experience with UV irradiation for virus inactivation. Another promising option, not available in egg-based processes, would be the generation of cell lines, which stably express PB2, and thus, complement missing viral functions for DI244 production. The use of such a cell line would probably also minimize the risk of unwanted accumulation of other defective (interfering) particles and allow better designed studies to elucidate the mechanisms of DIP replication, not only in cell cultures, but also in animals and humans to explore the full potential of DIPs as antivirals. However, while such an approach would eliminate the need to use infectious STV for seed generation as well as avoid the establishment of inactivation processes, scale-up, and, in particular, optimization of cell-specific DIP yields could be issues to be addressed.
